# The Effects of Gamified Virtual Reality on Muscle Strength and Physical Function in the Oldest Old—A Pilot Study on Sarcopenia-Related Functional Outcomes

**DOI:** 10.3390/jcm15020621

**Published:** 2026-01-13

**Authors:** Żaneta Grzywacz, Justyna Jaśniewicz, Anna Koziarska, Joanna Macierzyńska, Edyta Majorczyk

**Affiliations:** 1 Faculty of Production Engineering and Logistics, Opole University of Technology, 76 Prószkowska St., 45-758 Opole, Poland; 2 Center of Education Applications of Mathematic, Opole University of Technology, 76 Prószkowska St., 45-758 Opole, Poland; 3 Faculty of Physical Education and Physiotherapy, Opole University of Technology, 76 Prószkowska St., 45-758 Opole, Poland

**Keywords:** exergaming, immersive exercise, geriatric rehabilitation, functional fitness, age-related functional decline, long-term care residents, digital health in aging

## Abstract

**Background/Objectives:** Sarcopenia is an age-related decline in muscle mass and strength, reducing mobility and functional independence and increasing the risk of falls. Non-pharmacological interventions remain the most effective strategies to prevent or delay its progression, with exercise recognized as the primary approach. Virtual reality (VR)-based training has recently emerged as a promising tool to promote physical activity; however, its application among the oldest-old individuals remains underexplored. This is a randomized controlled pilot study to evaluate the effects of VR-based intervention using the game “Beat Saber” on muscle strength and selected physical performance indicators related to sarcopenia risk in older adults. **Methods:** Thirty-eight residents (mean age: 87.2) of a long-term care facility were randomly assigned to either a VR group or a control group. The VR group participated in 12 supervised VR-based training sessions of 20 min per session, three times per week for four weeks. Handgrip strength, the arm curl test, 30-s chair stand, a 2-min step-in-place test, and an 8-foot up-and-go test were assessed before and after the intervention. **Results:** Linear mixed-model analyses revealed significant group-by-time interactions for upper- and lower-limb strength (handgrip, arm curl, chair stand; *p* < 0.05), favoring the VR group. Agility and endurance (8-foot up-and-go, 2-min step-in-place) showed no significant interactions. In the VR group, the 30-s chair stand performance correlated positively with the arm curl and the 2-min step-in-place tests results, while handgrip strength correlated with the arm curl performance. In the control group, the 30-s chair stand test results correlated strongly with the 8-foot up-and-go and 2-min step-in-place tests, but no significant correlations were found for handgrip strength. **Conclusions:** The findings indicate short-term functional benefits of VR exercise among the oldest-old adults. VR-based training appears to be an effective and well-tolerated method to enhance physical performance in individuals aged 80 and older and may represent a valuable strategy for improving functional performance indicators associated with sarcopenia risk in adults aged 80 years and older.

## 1. Introduction

With the progressive aging of populations worldwide and the increasing prevalence of geriatric syndromes, greater attention is being given to the health challenges faced by the elderly, including the need for care, support and medical intervention. Thus, within aging populations, an increasing proportion of individuals aged 80 years and older, commonly defined as the oldest-old, present with substantial functional limitations and heightened vulnerability.

Among the conditions associated with aging sarcopenia stands out as a particularly common and serious issue, representing a significant public health challenge [1]. Officially recognized as a disease in 2016, sarcopenia is a syndrome characterized by the gradual loss of muscle mass and strength, which leads to reduced mobility, a higher risk of falls and a loss of functional independence [2,3]. As key aspects of sarcopenia, such as muscle quantity and quality, remain challenging to assess accurately in clinical practice, the diagnosis is typically based on multiple parameters. According to the revised guidelines of the European Working Group on Sarcopenia in Older People (EWGSOP2), the diagnostic process involves the assessment of muscle strength (e.g., handgrip strength or chair stand test), confirmation by evaluating muscle mass or quality, and, when applicable, the evaluation of physical performance. Moreover, EWGSOP2 recommends calf circumference (CC) and mid-upper-arm circumference (MUAC) as practical screening tools. The use of clearly defined cut-off points for each variable allows for the identification and staging of sarcopenia [4]. For example, the CC of ≤31 cm is commonly used as a threshold for sarcopenia. In the case of MUAC, values <22 cm for women and <23 cm for men are indicative of potential muscle mass loss.

Elderly people are particularly at risk of sarcopenia, as their natural ability to regenerate and adapt physically is limited [1,5,6,7]. Sarcopenia is estimated to affect 5% to 13% of individuals over the age of 60, with prevalence increasing to 50% among those aged 80 and older [8]. The main risk factors for development of sarcopenia include a lack of regular physical activity, chronic inflammation, metabolic diseases and nutritional deficiencies, particularly inadequate intake of protein and vitamin D [4,9,10].

Currently, no effective pharmacological treatments are available for sarcopenia, and non-pharmacological interventions remain the most appropriate and effective strategies to prevent or delay its progression [11], with exercise recognized as the primary intervention. The key challenge, therefore, lies in motivating older adults to engage in physical activity. In response to this issue, games in virtual reality (VR), particularly those that incorporate motor and cognitive components, offer a promising avenue for supporting older adults [12]. Supervised exercise programs incorporating gamification have been shown to increase physical activity levels in individuals with chronic conditions when compared to standard exercise. This approach may enhance engagement in physical activity, provided that appropriate supervision is ensured. However, further high-quality studies and subgroup analyses are needed to fully evaluate the long-term impact of gamified interventions in patients with chronic diseases [13].

Unfortunately, older adults are among the populations most affected by digital exclusion, due not only to technical infrastructure limitations, but more importantly to negative beliefs and stereotypes that perpetuate this exclusion [14]. Games are often perceived as the domain of younger individuals and may be considered unattractive or irrelevant to the elderly. Nonetheless, emerging evidence suggests that the use of VR-based technologies can positively influence the physical, cognitive, and mental health of the elderly [15,16]. Despite increasing interest in VR-based rehabilitation for older adults, high-quality evidence remains scarce, particularly for the oldest-old (≥80 years). Most studies focus on younger seniors (60–75 years), limiting the applicability of findings to older age groups [17,18]. Existing evidence suggests that VR may improve balance and gait but not lower limb strength [17], and that VR-based training can benefit activities of daily living, frailty, and mental health [18]. However, most interventions do not use widely available commercial games. While titles such as Beat Saber show promise in younger adults [19], no randomized controlled trials have examined their effects in those ≥80 years. Consequently, the effectiveness of VR in both preventing and treating sarcopenia among the oldest-old remains uncertain, and robust evidence on its long-term impact is lacking.

Therefore, this study was designed as a randomized controlled pilot trial to evaluate the effects of a rhythm-based virtual reality intervention on physical performance parameters related to sarcopenia risk in the oldest-old population (≥80 years). The primary hypothesis was that short-term VR-based training would result in greater improvements in upper-limb muscle strength compared with standard institutional physical activity. The primary outcome was handgrip strength. Secondary outcomes included the arm curl test, lower-limb functional performance, aerobic endurance, agility, and dynamic balance.

## 2. Materials and Methods

### 2.1. Participants

This study is a secondary analysis using data from a previously conducted randomized controlled trial described by Grzywacz et al. (2025) [20]. The primary aim of the original study was to assess the effects of virtual-reality-based gaming on visuomotor coordination and attention in the oldest-old population. The present secondary analysis was pre-planned to examine a different set of outcomes derived from the same intervention and cohort, specifically functional performance indicators related to sarcopenia risk.

The cohort consisted of 38 long-term residents of a nursing home in Opole (29 women and 9 men), all aged 65 or older, who were randomized by sex into two groups (VR group and control group) in a 1:1 ratio using the Research Randomizer tool (https://www.randomizer.org/, accessed on 19 July 2024), with stratification by sex to ensure balanced distribution of women and men between groups. The randomization sequence was generated prior to group allocation, and assignment was performed after participant enrollment.

Participants were exposed to comparable daily routine activity schedules and meal provision, as physical activity and dietary plans were institutionally standardized for all residents and monitored by facility staff. Daily activity levels and dietary intake were not quantitatively assessed using questionnaires, dietary records or wearable devices. Therefore, these factors were controlled at the organizational level rather than measured directly. All participants continued participation in the facility’s standard physical activity program, which focused on maintaining general mobility and motor coordination. Participants in the VR group also engaged in virtual-reality-based gaming, as detailed in the Intervention section.

Anthropometric parameters relevant to the assessment of sarcopenia were assessed using standardized procedures, as described below. Calf circumference was measured at the widest part of the dominant calf without compressing the skin or underlying muscle. Participants were seated comfortably with knees flexed at 90 degrees and feet flat on the floor. Mid-upper-arm circumference was measured on the non-dominant arm at the midpoint between the acromion and olecranon processes. Participants remained seated with the arm relaxed alongside the body. All measurements were performed using a non-stretchable tape, and two readings were taken for each site and averaged if the difference between them was ≤0.5 cm. Final values were recorded to the nearest 0.1 cm.

The study protocol was approved by the Bioethics Committee of the Hirszfeld Institute of Immunology and Experimental Therapy (approval number: KB-10/2024), and conducted in accordance with the ethical principles outlined in the Declaration of Helsinki. All participants signed an informed consent form prior to enrolment.

This pilot study was retrospectively registered (ClinicalTrials.gov Identifier: NCT06864325). The registration occurred after data collection due to the exploratory, feasibility-oriented nature of the project.

Additionally, seniors were qualified to this study through physician assessment of general health and screening for contraindications to VR headset use. Eligibility criteria required participants to be 60 years of age or older, in generally good physical condition, and willing to provide written informed consent for participation. Individuals were excluded if consent was not granted or if contraindications to the use of a VR headset were present. These included temporary conditions such as fatigue or exhaustion, drowsiness, nausea, anxiety or stress, as well as acute illnesses (e.g., cold or flu), and chronic conditions, including active cancer undergoing intensive treatment, inability to perform physical exercise, visual or hearing impairments preventing the use of VR goggles, or cognitive disorders that limited comprehension of exercise instructions or cooperation with the research team.

### 2.2. Study Design—Intervention

**Figure 1:**
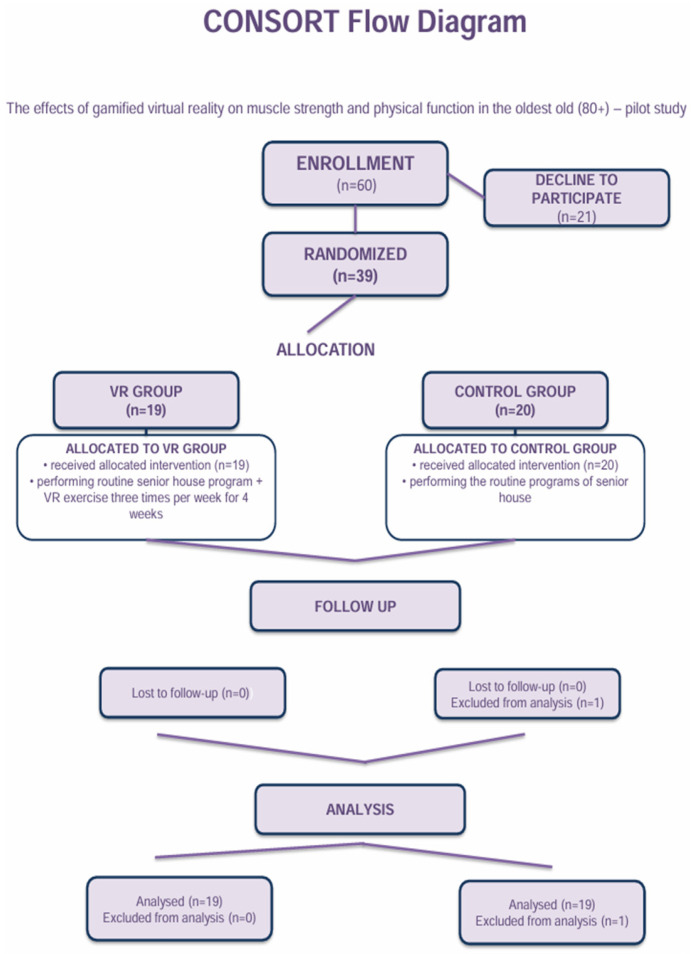
CONSORT flow diagram showing participant enrollment, randomization, allocation, follow-up, and analysis (*n* = 39 randomized).

This study followed the CONSORT 2010 guidelines, with participant enrollment and allocation shown in Figure 1. The VR intervention protocol was identical to that described in our previous publication [20]. For this secondary analysis, the same study design was used. Briefly, participants randomly allocated to the VR group completed a 4-week virtual reality exercise program using the commercial rhythm game Beat Saber, delivered via a Meta Quest 2 head-mounted display (Meta Platforms, Inc., Menlo Park, CA, USA). Beat Saber is a commercial virtual reality game developed by Beat Games and distributed by Meta Platforms, Inc., and was used under standard consumer conditions without any modification in software or content. The system used integrated inside-out 6DoF tracking, and participants held two motion controllers, each weighing approximately 150 g. Training was performed in a seated position within a safe 1.5 m × 1.5 m area. The game was set at the “Easy” difficulty level for all participants, with the “No Arrows” and “No Walls” modifiers activated to reduce cognitive and balance demands. The difficulty level remained constant throughout the intervention, as the primary aim was safety and feasibility assessment rather than progressive overload. Sessions lasted approximately 20 min and were conducted three times per week. The program emphasized upper-limb movements synchronized with audiovisual stimuli and was supervised by a physiotherapist to ensure safety and appropriate adaptation to each participant’s functional capacity. Participants assigned to the control group continued their routine physical activity program provided by the institution. The program aimed to maintain general physical function and coordination and was tailored to the functional capacities of older residents. Sessions typically consisted of preparatory movements, light whole-body exercises using basic equipment (e.g., elastic bands, sticks, or light hand weights), followed by simple coordination tasks such as rhythm-based movements or ball exercises, and concluded with stretching and relaxation components. All sessions were supervised by a physiotherapist and incorporated elements supporting social interaction. The control group intervention did not include virtual reality exposure or gamified elements and represented routine care provided by the institution. Both the VR and control interventions were delivered over the same 4-week period, with comparable session frequency and duration, and were supervised by a physiotherapist to ensure participant safety.

**Figure 2:**
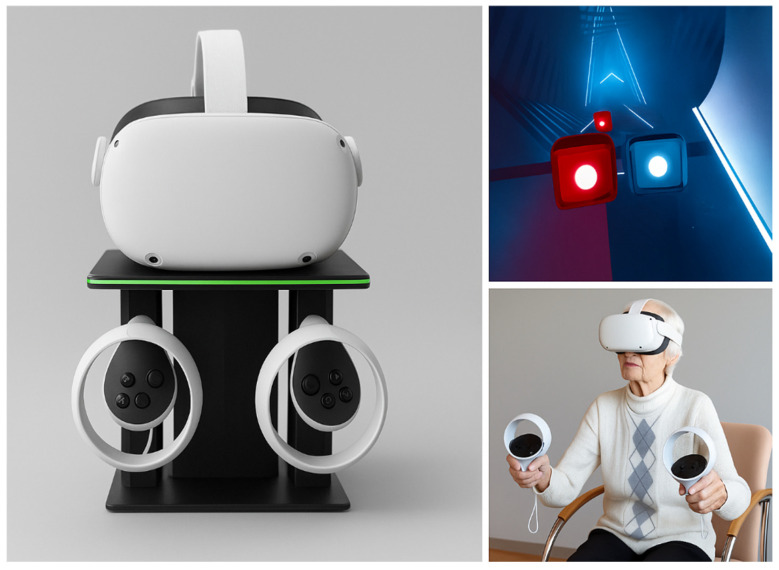
A composite visualization of the virtual reality (VR) training setup used in this study, illustrating the headset, controllers, and the experimental environment of the Beat Saber game. Source: The composite image was created by the authors using their own photographs with a screenshot from Beat Saber (© Beat Games/Meta Platforms Inc., Menlo Park, CA, USA), integrated and stylized with AI-assisted tools (ChatGPT/DALL·E, OpenAI, 2025).

A visual representation of the training setup and game interface is provided in Figure 2.

### 2.3. Outcomes Measures

All assessments were conducted at two time points: baseline (T_0_) and after the completion of the 4-week intervention period (T_1_; approximately 20 min after the last session). The measurements focused on parameters related to sarcopenia risk, such as an upper-limb strength and physical performance.

#### 2.3.1. Muscle Strength (Handgrip Strength)

Muscle strength was assessed using the MG-4800 digital medical hand dynamometer (Shenzhen Camry Electronic Co., Ltd., Shenzhen, China) to measure isometric handgrip force. The device, equipped with a 20 kg resistance spring, provided reliable and accurate measurements within the strength range of older adults. Participants were seated on an armless, straight-backed chair with feet flat on the floor. The test was performed on the dominant hand with the elbow flexed at 90°, forearm in a neutral position (thumb up), and wrist in slight extension, following standardized handgrip testing procedures. The dynamometer grip was adjusted to match the participant’s hand size for optimal positioning and ergonomics. Participants were instructed to squeeze the handle with maximum effort for 3–5 s while avoiding trunk or shoulder compensation. At both time points (T_0_ and T_1_), three trials were performed with at least 30 s of rest between attempts. The final score was calculated as the average of the three trials. Although the Jamar dynamometer is commonly considered the gold standard for handgrip strength assessment, the same device was used consistently across all participants and time points. Therefore, the handgrip strength results were interpreted primarily in terms of within-group and between-group changes rather than absolute normative comparisons.

#### 2.3.2. Functional Fitness Battery

Physical performance was assessed using four tests from the Senior Fitness Test (SFT) protocol: the 30-s chair stand test (lower body strength), the arm curl test (upper-limb muscular endurance; 2 kg dumbbell for women, 3 kg for men), the 2-min step-in-place test (aerobic capacity; number of right-leg steps recorded), and the 8-foot up-and-go test (agility and dynamic balance). These measures were selected as potential indicators of sarcopenia. Detailed testing procedures followed the standardized methodology as described previously [21]. All assessments were conducted by trained staff under standardized conditions and repeated at baseline (T_0_) and post-intervention (T_1_) to evaluate functional changes.

#### 2.3.3. Statistical Analyses

Demographic and anthropometric characteristics of the participants, including age, height, weight, and BMI, were analyzed using descriptive statistics. The Shapiro–Wilk test was applied to assess the normality of data distribution. To assess the effects of the intervention, linear mixed models (LMMs) were applied with time (T_0_ and T_1_), group (VR group, control group), and their interaction (time × group) as fixed factors, and participants (ID) as a random intercept. This approach accounted for the repeated-measures structure of the data and individual variability. The significance of fixed effects was tested using the Satterthwaite test with Type III sums of squares. Estimated marginal means and 95% confidence intervals were reported for each condition. To assess the strength and direction of associations between measured outcomes, Spearman’s rank correlation coefficient (rho) was calculated. This non-parametric method was used due to non-normal distributions of the certain analyzed data. *p*-values ≤ 0.05 were considered statistically significant. Statistical analyses were performed using Statistica v.13 (StatSoft, Inc., Tulsa, OK, USA) and the JAMOVI statistical software (version 2.6.26) for specific paired comparisons and effect size calculations. No a priori sample size calculation was performed, as this study was designed as a pilot and feasibility-oriented investigation.

## 3. Results

For anthropometric characteristics of the study groups, as well as recorded baseline anthropometric parameters relevant to the assessment of sarcopenia (calf and mid-upper-arm circumferences, see Table 1.

The presented results were collected from participants in accordance with the CONSORT flow Grzywacz et al., 2025 [20]. No dropouts, adverse events, or intervention-related complications were observed in either group during the study period. The measures related to sarcopenia risk were chosen among widely used indicators: a handgrip strength test and functional fitness battery tests such as arm curl, 30-s chair stand, 8-foot up-and-go, and 2-min step-in-place. All parameters were assessed at two time points: prior to the intervention (T_0_) and following the intervention (T_1_). All participants were divided into two groups: the VR group (*n* = 19) and the control group (*n* = 19). For certain analyzed data, non-normality of distributions were observed; thus, central tendency is reported using both median with Q_1_–Q_3_ quartiles range and mean with standard deviation (Table 2). Muscle-mass-related anthropometric parameters were assessed at baseline and post-intervention; however, no statistically significant changes were observed. Due to the short duration of the intervention, the outcomes were interpreted primarily as functional performance indicators associated with sarcopenia risk rather than as evidence of structural muscle adaptations or sarcopenia diagnosis.

These thresholds were derived from validated criteria reported in previous studies and international guidelines (EWGSOP2, 2019, [4]; Lima et al., 2023 [22]; Sawada et al., 2021 [23]; Martinez et al., 2015 [24]; Poncumhak et al., 2023) [25] and indicate functional performance levels associated with an increased risk of sarcopenia rather than a con-firmed clinical diagnosis. The cut-off points used to classify participants as being at risk of sarcopenia, together with the corresponding references, are presented in Table A1 (Appendix A).

Linear mixed-model analyses were performed to examine the effects of the VR-based intervention on muscle strength and physical performance over time (Table 2). For handgrip strength, the main effects of time (F = 0.10, *p* = 0.754) and group (F_1_ = 0.22, *p* = 0.643) were not significant; however, the time × group interaction reached significance (F = 4.68, *p* = 0.037). Estimated marginal means indicated that handgrip strength showed a small increase in the VR group (17.93 kg at T_0_ and 18.69 kg at T_1_), while it declined slightly in controls (17.82 and 17.25 kg, respectively at T_0_ and T_1_), suggesting a small protective effect of the VR intervention. For the arm curl test, significant main effects of time (F = 10.16, *p* = 0.003) and group (F = 18.70, *p* < 0.001) were observed, together with a significant time × group interaction (F = 6.57, *p* = 0.015). The VR group improved markedly (14.16 to 18.53 repetitions), whereas the control group showed only a minimal change (T_0_: 10.32 and T_1_: 10.79). In the 30-s chair-stand test, all effects were significant: time (F = 21.21, *p* < 0.001), group (F = 23.64, *p* < 0.001), and time × group (F = 22.67, *p* < 0.001). Performance in the VR group increased substantially (9.32 to 12.47 repetitions), while no meaningful change occurred in the controls (4.16 and 4.11, respectively at T_0_ and T_1_). For the 8-foot up-and-go test, neither the main effects nor the interaction were significant (time, *p* = 0.165; group, *p* = 0.657; time × group, *p* = 0.301), indicating comparable agility performance across groups. Finally, for the 2-min step-in-place test, significant main effects of time (F = 6.84, *p* = 0.013) and group (F = 7.43, *p* = 0.010) were found, but the interaction was non-significant (*p* = 0.990). Both groups improved slightly over time, though the VR group maintained higher overall step counts (T_0_: 74.7 and T_1_: 85.8 steps) compared to controls (42.2 to 53.2 steps).

Overall, the mixed-model results demonstrate significant group-by-time interactions for upper- and lower-limb strength (handgrip, arm curl, chair stand), supporting the effectiveness of the VR intervention in enhancing functional performance among the oldest-old participants.

**Figure 3:**
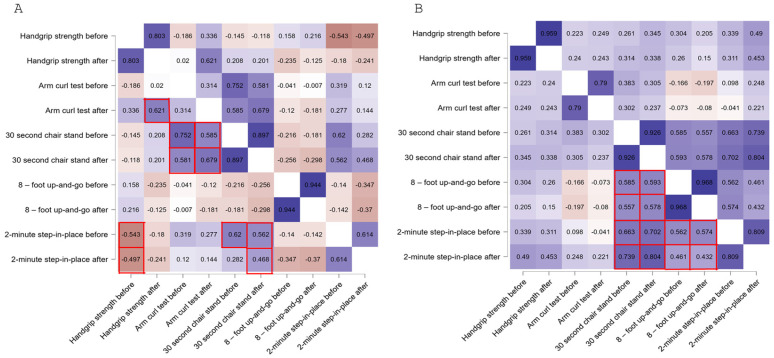
Spearman’s rank correlation matrix between sarcopenia-related functional indicators before and after the intervention in the VR group (**A**) and the control group (**B**). Colors represent the strength and direction of correlations, with blue indicating positive correlations and red indicating negative correlations; color intensity reflects the magnitude of the correlation coefficient. The red boxes highlight statistically significant correlations discussed in detail in the text.

Moreover, Spearman’s correlations coefficients were calculated to illustrate the relationships between all variables indicative of sarcopenia risk. In Figure 3, squares outlined in red indicate statistically significant correlations between different parameters (*p* < 0.05), with their strength and direction of the associations represented by color saturation (warm hues indicating positive correlations and blue hues indicating negative correlations). To facilitate the analysis, statistically significant relationships for the same parameters measured at two time points (T_0_ and T_1_) were not marked in the figure. In the VR group, the 30-s chair stand test showed moderate to strong positive correlations with both the arm curl and the 2-min step-in-place tests results, measured before and after intervention. Specifically, the 30-s chair stand at T_0_ correlated positively with the arm curl tests at both T_0_ and T_1_, as well as with the 2-min step-in-place test at T_0_. Similarly, the 30-s chair stand at T_1_ correlated positively with both the arm curl and the 2-min step-in-place tests at T_0_ and T_1_. Additionally, handgrip strength at T_0_ correlated negatively with both T_0_ and T_1_ results of the 2-min step-in-place tests, whereas handgrip strength at T_1_ showed a positive correlation with the arm curl test at the same time point. In the control group, several significant positive correlations were observed among some physical performance measures, primarily at the time point before the intervention. The 30-s chair stand test at both T_0_ and T_1_ showed strong positive correlations with the 8-foot up-and-go at T_0_ and T_1_, as well as with the 2-min step-in-place test at T_0_ and T_1_. Furthermore, the 8-foot up-and-go at T_0_ and T_1_ and the 2-min step-in-place test at T_0_ and T_1_ positively correlated among themselves. In contrast to the VR group, no significant correlations were observed between handgrip strength and other parameters in the control group.

## 4. Discussion

The aim of this pilot study was to evaluate the potential of the commercial VR game Beat Saber to improve functional performance indicators associated with sarcopenia risk in the oldest-old population. The results indicate that a 4-week virtual-reality-based training can significantly improve selected physical fitness parameters related to sarcopenia risk in elderly individuals. The most notable improvements were observed in upper limb strength (arm curl test) and lower limb endurance (30-s chair stand test). In the VR group, handgrip strength remained stable over the course of intervention, whereas a decline was observed in the control group. Moreover, our findings suggest that, within the VR group, baseline muscle strength and physical performance were interrelated and improvements in handgrip strength corresponded with better functional outcomes following the intervention. In contrast, the control group exhibited fewer dynamic changes across the same measures, suggesting that standard physical activity alone may be less effective in enhancing short-term functional fitness in this population. It should be noted that the modest decline observed in handgrip strength in the control group likely reflects short-term biological and measurement variability commonly reported in very old adults, rather than a true or clinically meaningful deterioration in muscle function over such a brief period [26].

Overall, both the VR and control groups showed a reduced frequency of sarcopenia indicators based on established score cut-off points; however, the reduction was more pronounced in the VR group. In particular, we employed the widely recognized handgrip strength test, which is considered a strong predictor of overall health and mortality in older adults [27]. The test results remained stable in the VR group but declined in the control group, suggesting a potential protective effect of VR-based training. Our intervention shared some characteristics with low-intensity, resistance-like upper-limb activity through the use of the Beat Saber game, in which the mass of the controllers and rapid, rhythmic arm movements likely provided dynamic resistance. Even low-intensity resistance-type activity, when delivered in an engaging format, can yield measurable functional benefits. Previous research shows that moderate, resistance-based exercise performed regularly can improve grip strength and physical function [28,29,30] but has limited effects on muscle mass [31]. In contrast, traditional resistance and multicomponent exercise programs, considered the cornerstone of sarcopenia management, typically rely on structured protocols and progressive overload and have demonstrated robust improvements in muscle strength and functional performance in older adults [32]. In contrast, the present VR-based intervention was not designed to elicit comparable physiological training responses but rather to examine short-term functional feasibility in the oldest-old population. It is possible that our VR-based intervention primarily induced early neuromuscular adaptations such as improved motor unit recruitment and efficiency before measurable hypertrophy occurred, consistent with the phenomenon described by Song et al. (2023) [33]. Moreover, for assessment of measures related to sarcopenia risk, we employed a battery of functional fitness tests, consisting of several simple but effective measures that provide a broad evaluation of physical performance, including lower body strength, upper-limb muscular endurance, aerobic capacity, agility and dynamic balance Accordingly, improvements in selected functional outcomes related to endurance and dynamic task performance (2-min step-in-place, 8-foot up-and-go) observed in the VR group suggest potential benefits in movement coordination and task execution. However, no consistent improvements were observed in agility-specific measures. This finding is consistent with previous studies indicating that improvements in agility and rapid directional changes typically require task-specific, weight-bearing, and progressively challenging training stimuli, particularly in very old populations [34,35].

In this paper, a comprehensive evaluation consists of correlations between analyzed components of physical fitness, which are often interrelated [36,37]. Our model revealed, in the VR group, consistent moderate-to-strong correlations between lower-body strength, upper-limb endurance, and aerobic capacity both before and after the intervention. In contrast, the control group showed significant associations between functional measures, which were observed mainly at baseline, with no notable correlations involving handgrip strength. These findings suggest that gamified VR training may enhance multiple interrelated components leading to more integrated functional gains to mitigate sarcopenia-related decline and promote physical independence in the oldest-old population. This potential benefit of VR-based interventions is consistent with previous evidence that yields multidimensional functional improvements in older adults, including strength, balance and aerobic capacity, as well as cardiovascular performance [38,39,40,41,42,43,44,45]. Moreover, prior evidence links virtual environments with improved motor learning and cognitive–emotional outcomes in older adults [41,42,43]. Our earlier findings with the same intervention further confirm benefits for visuomotor coordination and attention [20]. On the other hand, it seems that without targeted intervention, the overall functionality of the elderly may remain more difficult to improve.

Physical function naturally declines owing to physiological changes associated with aging, such as sarcopenia. In the present study, participants in the VR group showed significant improvements in key physical performance outcomes, indicating that VR-based exercise may offer substantial benefits for the health and quality of life of older adults. Therefore, VR-based training may represent a valuable strategy for supporting functional performance and physical capacity in elderly at risk of sarcopenia. It is of particular importance in sustaining engagement among the oldest-old that, unlike conventional exercise, VR offers immersion, enjoyment, and rewards that enhance intrinsic motivation. Although VR is often associated with younger users [14], our results show that it can be effectively adapted for adults aged 80 years and older. High acceptance rates, despite concerns about digital exclusion and cognitive limitations, likely stemmed from the simple, rhythmic, and feedback-driven format, which encouraged engagement and adherence.

## 5. Limitations

Although muscle-mass-related anthropometric parameters were assessed before and after the intervention, no statistically significant changes were observed. This is most likely attributable to the short duration of the intervention, which was insufficient to induce measurable structural muscle adaptations. Consequently, the present findings should be interpreted as short-term functional responses rather than evidence of changes in muscle mass or sarcopenia as a clinical condition. Moreover, handgrip strength was assessed using a digital dynamometer other than the Jamar device, which may limit direct comparability with studies using gold-standard instrumentation; however, internal consistency was ensured by using the same device and protocol throughout this study. Although the results suggest a beneficial impact of VR-based intervention on selected physical performance parameters in the oldest-old population, this study was designed as a pilot study feasibility-oriented investigation and is therefore subject to several methodological limitations. Most notably, no a priori sample size calculation was performed, and the relatively small sample size (*n* = 38) determined by the available population of the long-term care facility limits statistical power and restricts the generalizability of the findings to broader elderly populations. Consequently, the results should be interpreted with caution, particularly with regard to the detection of smaller effects. Despite these limitations, this study demonstrates the practical feasibility of implementing VR-based exercise interventions in institutionalized very old adults and provides preliminary data that may inform future sample size and power calculations in larger, confirmatory trials. Furthermore, all participants were recruited from a single long-term care facility, which narrows the social and environmental context of this study and may further limit external validity.

Daily physical activity levels and dietary intake were not objectively quantified using questionnaires, dietary records, or wearable devices. Although daily routines and meal provision were standardized at the institutional level, unmeasured individual variability in activity and nutrition may have influenced the observed outcomes. The trial was registered retrospectively, reflecting its exploratory and feasibility-oriented design. The intervention lasted only four weeks and all outcome measures were collected immediately post-intervention. Post-intervention assessments were conducted shortly after the final training session, which may have been influenced by acute exercise effects, including transient fatigue, potentially affecting performance outcomes. No follow-up was conducted to assess the long-term sustainability of the observed effects, such as maintenance of muscle strength gains or functional performance improvements. The absence of longitudinal data prevents the determination of whether the benefits are transient or can be sustained in daily life. Minor baseline imbalances between groups were observed but were not statistically adjusted for due to the small sample size and exploratory nature of the analysis. In future trials, a larger sample with stratified randomization and covariate adjustment is recommended to enhance statistical validity. It is also worth considering the integration of dietary components into future activation programs, particularly nutritional strategies based on low-carbohydrate or anti-inflammatory diets, which, according to the literature, may support sarcopenia management [44,45]. A comprehensive approach combining exercise, nutrition and modern technologies could offer a more effective strategy to delay age-related functional decline and maintain independence in older adults.

## 6. Conclusions

This pilot study demonstrated that a four-week gamified virtual reality training using the commercial game Beat Saber can improve key indicators of physical function among the oldest-old population. Significant gains were observed in both upper- and lower-limb strength, particularly in arm curl and chair stand performance. Notably, handgrip strength was preserved in the VR group but declined in the control group, highlighting the potential of such interventions to slow functional deterioration in advanced age.

The results support the feasibility and acceptability of VR-based exercise programs in institutionalized adults aged 80 years and older. The use of immersive and engaging environments may facilitate adherence and participation, addressing common barriers to physical activity in this population. Nevertheless, the findings should be interpreted in light of the pilot and feasibility-oriented design of this study, the limited sample size, and the absence of long-term follow-up.

Future studies should involve larger samples, a longer intervention period, follow-up assessments and integrative approaches combining VR-based physical training with targeted dietary strategies (e.g., anti-inflammatory or protein-rich diets). Such a multifaceted model may represent a promising pathway toward preserving autonomy and quality of life in aging populations by improving functional performance indicators associated with sarcopenia risk.

## Figures and Tables

**Table 1:** Characteristics of study groups.

Groups (*n*)	Age (Years)	Sex (Women/Men)	Weight (kg)	Height (cm)	BMI (kg/m^2^)
All cases (38)	87.2 (±6.3)	29/9	68 (±14.5)	157.4 (±10.2)	27.7 (±6.4)
VR group (19)	88.0 (±6.4)	14/5	70.8 (±8.3)	161.4 (±8.3)	27.2 (±6.6)
Control group (19)	86.4 (±6.3)	15/4	65.1(±12.2)	153.5 (±10.6)	28.2 (±6.4)

**Table 2:** Intergroup and intragroup differences in measures related to sarcopenia risk.

Parameters Related to Sarcopenia Risk		Groups			
		VR Group T_0_ (Before)	VR Group T_1_ (After)	Control Group T_0_ (Before)	Control Group T_1_ (After)
**Handgrip strength** **(kg)**	Mean (SD)	17.9 (5.9)	18.7 (5.6)	17.8 (4.36)	17.2 (4.9)
	Median (Q_1_-Q_3_)	16.8 (15.2–18.5)	17.9 (15.2–20.1)	16.4 (14.9–20.9)	15.2 (13.8–20.7)
	Difference T_0_ vs. T_1_	0.8 ↑		0.6 ↓	
	Sarcopenia risk (*n*;%) ^#^	8 (42.1)	7 (36.8)	14 (73.7)	13 (68.4)
**Arm curl** **test (*n*)**	Mean (SD)	14.2 (5.6)	18.5 (5.0)	10.3 (4.4)	10.8 (3.8)
	Median (Q_1_–Q_3_)	14.0 (11–19.5)	18.0 (14.5–22.5)	9.0 (7.5–13)	11.0 (8–12.5)
	Difference T_0_ vs. T_1_	4.3 ↑		0.5 ↑	
	Sarcopenia risk (*n*;%) ^#^	6 (31.6)	1 (5.3)	13 (68.4)	10 (52.6)
**30-s chair stand (*n*)**	Mean (SD)	9.32 (4.0)	12.5 (5.5)	4.16 (3.7)	4.11 (4.3)
	Median (Q_1_–Q_3_)	9.0 (8.0–12.0)	14.0 (9.5–16.5)	3.0 (2.0–5.5)	3.0 (0.0–7.0)
	Difference T_0_ vs. T_1_	5.0 ↑		0.0 =	
	Sarcopenia risk (*n*;%) ^#^	19 (100.0)	14 (73.7)	19 (100.0)	19 (100.0)
**8-foot up-and-go (*n*)**	Mean (SD)	18.7 (16.2)	15.9 (10.7)	15.3 (16.2)	14.9 (16.5)
	Median (Q_1_–Q_3_)	15.5 (9.02–17.9)	12.5 (9.43–19.1)	16.2 (0.0–26.6)	16.5 (0.0–26.9)
	Difference T_0_ vs. T_1_	2.8 ↓		0.4 ↓	
	Sarcopenia risk (*n*;%) ^#^	5 (26.3)	6 (31.6)	9 (47.4)	7 (36.8)
**2-min step-in-place (*n*)**	Mean (SD)	74.7 (33.2)	85.8 (30.7)	42.2 (41.5)	53.2 (48.8)
	Median (Q_1_–Q_3_)	75.0 (50.0–97.5)	94.0 (73.0–106.0)	41.5 (0.0–83.5)	48.4 (0.0–91.0)
	Difference T_0_ vs. T_1_	11.1 ↑		11.0 ↑	
	Sarcopenia risk (*n*;%) ^#^	8 (42.1)	2 (10.5)	12 (63.2)	10 (52.6)
	**Statistical Analyses—Linear Mixed Model**				
	**Time/Group**	**EMM (95%CI)**	**Effect**	**F_1,36_**	***p* Value**
**Handgrip strength** [kg]	T_0_ VR	17.9 (15.6–20.3)	Time	0.100	0.754
	T_1_ VR	18.7 (16.4–21.0)	Group	0.219	0.643
	T_0_ control	17.8 (15.5–20.2)	Time × group	4.684	0.037
	T_1_ control	17.2 (14.9–19.6)			
**Arm curl** **test (*n*)**	T_0_ VR	14.2 (12.0–16.3)	Time	10.161	0.003
	T_1_ VR	18.5 (16.4–20.7)	Group	18.701	<0.001
	T_0_ control	10.3 (8.2–12.4)	Time × group	6.574	0.015
	T_1_ control	10.8 (8.7–12.9)			
**30-s chair stand (*n*)**	T_0_ VR	9.3 (7.3–11.3)	Time	21.211	<0.001
	T_1_ VR	12.5 (10.5–14.5)	Group	23.641	<0.001
	T_0_ control	4.2 (2.2–6.1)	Time × group	22.674	<0.001
	T_1_ control	4.1 (2.1–6.1)			
**8-foot up-and-go (*n*)**	T_0_ VR	18.7 (11.9–25.4)	Time	2.004	0.165
	T_1_ VR	15.9 (9.1–22.6)	Group	0.201	0.657
	T_0_ control	15.3 (8.6–22.1)	Time × group	1.103	0.301
	T_1_ control	14.9 (8.1–21.7)			
**2-min step-in-place (*n*)**	T_0_ VR	74.7 (57.1–92.2)	Time	0.100	0.754
	T_1_ VR	85.7 (68.2–103.4)	Group	0.219	0.643
	T_0_ control	42.2 (24.6–59.7)	Time × group	4.684	0.037
	T_1_ control	53.2 (35.6–70.7)			

Sarcopenia risk (*n*; %): ^#^ represents the proportion of participants whose performance in a given functional test was below the established cut-off values for muscle strength, endurance, mobility, or aerobic capacity.

## Data Availability

The data presented in this study are available on request from the corresponding author. The data are not publicly available due to the protection of research participant privacy.

## Appendix A

**Table A1:** Cut-off points for physical performance tests indicating sarcopenia risk (*n*; %) #.

Test	Cut-Off Point	Reference
Handgrip strength	<27 kg (men), <16 kg (women)	European Working Group on Sarcopenia in Older People 2 (EWGSOP2, 2019) [4]
Arm curl test	<11.5 repetitions (both men and women)	Lima, A.B., Baptista, F., Henriques-Neto, D., Pinto, A.A., & Gouveia, E.R. (2023) [22]
30-s chair stand test	<15 repetitions (women), <17 repetitions (men)	Sawada, S. et al. (2021) [23]
8-foot up-and-go test	>10.85 s	Martinez, B.P. et al. (2015) [24]
2-min step-in-place test	<60 steps	Poncumnhak, P. et al. (2023) [25]

Description of Sarcopenia Risk (*n*; %) #: The risk of sarcopenia among participants was determined based on their performance in selected functional fitness tests, applying established cut-off points from validated sources. According to the European Working Group on Sarcopenia in Older People 2 [4], low muscle strength was defined as handgrip strength <27 kg for men and <16 kg for women. Upper-limb muscular endurance was assessed using the arm curl test, with values below 11.5 repetitions considered indicative of reduced performance [22]. Lower-body strength was evaluated using the 30-s chair stand test, where fewer than 15 repetitions in women and 17 in men indicated reduced lower-limb function [23]. Functional mobility was assessed by the 8-foot up-and-go test, with times exceeding 10.85 s interpreted as reduced performance [24]. Aerobic endurance was measured using the 2-min step-in-place test, where the results below 60 steps indicated diminished functional capacity [25]. Participants who met one or more of these criteria were categorized as being at risk of sarcopenia. This multidimensional approach, integrating measures of muscle strength, endurance, mobility, and aerobic performance, provides a comprehensive assessment of age-related functional decline and identifies individuals potentially vulnerable to sarcopenia-related impairment.
